# Identification and functional analysis of lncRNAs and mRNAs between tumorigenesis and metastasis in CRC

**DOI:** 10.18632/aging.203775

**Published:** 2021-12-26

**Authors:** Hongtao Liu, Yuan Tian, Jiaxi Li, Guoxia Zhang, Qun Liu, Min Yang, Longtao Yue, Qiwei Cao, Guihui Zhang, Yuxia Cheng, Na Kong, Lei Fang, Shoupeng Li, Qing Sun

**Affiliations:** 1 Department of Pathology, The First Affiliated Hospital of Shandong First Medical University and Shandong Provincial Qianfoshan Hospital, Shandong Medicine and Health Key Laboratory of Clinical Pathology, Shandong Lung Cancer Institute, Shandong Institute of Nephrology, Jinan 250014, Shandong, P.R. China; 2 Somatic Radiotherapy Department, Shandong Second Provincial General Hospital, Shandong Provincial ENT Hospital, Huaiyin, Jinan 250023, Shandong, P.R. China

**Keywords:** long noncoding RNA, colorectal cancer, MIR29B2CHG93, tumorigenesis, metastasis

## Abstract

The role of long non-coding RNAs (lncRNAs) in colorectal cancer (CRC) tumorigenesis and metastasis remains poorly characterized. The aim of this study was to identify novel lncRNAs and their functions in CRC progression. Through microarray analysis of paired normal colorectal mucosa (NM), primary tumor (PT), and metastatic lymph node (MLN) tissues, lncRNA and mRNA expression patterns were identified. Further bioinformatic analyses were performed to compare the biological functions of lncRNAs between tumorigenesis and metastasis of CRC, which was further verified by TCGA-COAD and GSE82236. The expression of lncRNA MIR29B2CHG93 in paired CRC tissues was detected in a cohort of CRC patients. The effects of lncRNA MIR29B2CHG93 on proliferation, migration, and invasion were determined by *in vitro* experiments. We found that tumorigenesis-associated lncRNAs predominantly participated in the regulation of the EMT/P53/PI3K-Akt/KRAS signaling pathway as well as the processes related to cell cycle and cell mitosis, while metastasis-associated lncRNAs mainly regulated blood vessel morphogenesis and immune-related biological processes. Compared to the TCGA and GSE datasets, seven tumorigenesis-associated lncRNAs and eight metastasis-associated lncRNAs were identified. LncRNA MIR29B2CHG93 knockdown remarkably suppressed tumor growth and metastasis *in vitro*, which acted as a tumor promoter in CRC. The lncRNA MIR29B2CHG93 was significantly upregulated in CRC tissues and was indicator of unfavorable clinical outcome in CRC. These results revealed novel lncRNAs that provide new insights for an in-depth understanding of CRC progression. In particular, this study identified a novel lncRNA MIR29B2CHG93 in CRC progression, which might be a potential biomarker for diagnosis, prognosis and metastasis-prediction in CRC.

## INTRODUCTION

Colorectal cancer (CRC) is the most common gastrointestinal malignancy worldwide, and approximately 50% of patients with primary disease eventually develop metastatic disease [1]. It is widely accepted that the development of CRC is a multistage process, from normal mucosal epithelium to a malignant tumor and ultimately progresses to metastasis [2]. While the molecular mechanisms of CRC development have primarily focused on protein-coding genes to identify oncogenes and tumor suppressors and the role of microRNAs [3, 4], there is still a lack of studies focusing on lncRNAs in CRC development.

Long non-coding RNAs (lncRNAs) lack coding potential RNA molecules that are greater than 200 nucleotides in length [5]. LncRNAs are known to have diverse structural and regulatory roles in protecting chromosome integrity, maintaining genomic architecture, X chromosome inactivation, imprinting, transcription, translation, and epigenetic regulation [6]. lncRNAs serve as critical regulators of tumorigenesis and metastasis. For instance, (LOC105371049) regulates colorectal cancer proliferation, metastasis and metabolism [7], and LncRNA-FEZF1-AS1 tumor proliferation and metastasis in colorectal cancer by regulating PKM2 signaling [8]. However, the comprehensive characteristics of transcripts and genetic alterations from primary to metastatic disease remain limited in CRC.

Due to the lack of matched metastatic samples in The Cancer Genome Atlas Network (TCGA) [9], it is impossible for us to find novel lncRNAs correlated with metastasis through TCGA data. Advances in high-throughput DNA microarray analysis technology have provided an unbiased method for discovering the landscape of specific transcriptome alterations to metastasized tumors [10]. In view of the flaws of TCGA-COAD data and the advances in CRC, we conducted a comparative study between primary tumors (PT) and matched metastatic lymph nodes (MLN) to identify the differentially expressed lncRNAs and mRNAs associated with tumorigenesis and metastasis.

## MATERIALS AND METHODS

### Tissue samples

Human CRC primary tumor (PT) tissues and metastatic lymph nodes (MLN) and corresponding normal tissues were obtained from patients who underwent surgical resection at The First Affiliated Hospital of Shandong First Medical University from 2011 to 2018 were recruited. All patients provided written informed consent, and the study was approved by the ethical committee of The First Affiliated Hospital of Shandong First Medical University. Two experienced pathologists determined the histological type of CRC and the metastatic lymph nodes. Tumor staging was graded according to the TNM classification (AJCC 8th edition). Frozen and FFPE samples were collected for analysis.

### Cell culture and transfection

The human colorectal cancer cell lines SW480, HCT116, and Caco2 were obtained from the Cell Bank of the Chinese Academy of Sciences and were cultured in Dulbecco’s modified Eagle’s medium (Invitrogen, USA) containing 100 U/ml penicillin, 100 μg/ml streptomycin, and 10% fetal bovine serum (Invitrogen, USA) in a 5% CO2-humidified incubator at 37° C. shRNA lentiviruses co-expressing enhanced green fluorescent protein (EGFP) and shRNA against human MIR29B2CHG93 were purchased from Shanghai GeneChem Co., Ltd. (Shanghai, China). Scrambled shRNA (sh-NC) that targeted a non-specific sequence was used as a control. Cells were plated in 6-well plates at 1.5×105 cells per well, grown for 24 h, and then transfected with shRNA at low multiplicity of infection (MOI) for 24 h. Cells were harvested for analysis three days post-transfection.

### Microarray analyses

Total RNA was extracted using the TRIzol reagent (QIAGEN, Germany). The A260/A280 ratio of RNA was in the range of 1.9 to 2.1. Double-stranded complementary DNA(cDNA) was synthesized using Ambion® WT Expression Kit (Ambion, USA), labeled using the Affymetrix GeneChip® WT Terminal Labeling Kit (Affymetrix, USA), and then hybridized to the Affymetrix GeneChip Human Exon Array-Gminix lncRNA-WT v1.0 Microarray(Affymetrix, USA). After washing using the GeneChip® Hybridization Wash and Stain Kit (Affymetrix, USA), the slides were scanned using a GeneChip2 Scanner 3000 7G and the raw data were extracted from scanned images using Affymetrix Launcher (Affymetrix, USA). The microarray assay was conducted by the QiMing Biotechnology Company (Shanghai, China). Finally, the raw signal values from the microarray were log_2_ transformed to evaluate the expression levels of lncRNAs and protein-coding mRNAs. Significant differences in transcript abundance were determined using the paired t-test method. Transcripts with an absolute value of fold change >1.5 and P-value <0.05, were considered as differentially expressed between different groups.

### RNA extraction and quantitative real-time PCR analysis

Total RNA was extracted using TRIzol reagent (QIAGEN, Germany). RNA was reverse transcribed to cDNA using a reverse transcription kit (TaKaRa Biotechnology, Dalian, China). RT-PCR was performed using the SYBR green assay (TaKaRa Biotechnology) on an AB 7500 machine (Applied Biosystems Inc., USA). The SYBR primers used in this study were listed (Supplementary Table 7). GAPDH served as an internal control for normalization. Relative RNA abundance (fold change) of each lncRNA was calculated using the standard 2^−ΔΔCT^. The standard linear regression analysis (fold change of gene expression) was performed between microarray data and quantitative real-time PCR data using the calculated equation and correlation value (R^2^).

### 3-(4, 5-dimethylthiazol-2-yl)-2, 5-diphenyltetrazolium bromide (MTT) assay

Transfected cells were seeded at a density of 5000 cells/well in 96-well plates. After incubation, 20 μl of 5 mg/ml MTT was added to each well and incubated for another 4 h. Then, the supernatants were carefully removed, and 100 μl DMSO was added to each well. The proliferation curves were determined by calculating the relative value of absorbance measured at 570 nm using a microplate reader (Bio-Rad, USA).

### Transwell assay

Transwell assays were performed using Transwell chambers (pore size 8 μm; Costar Corporation, USA) with or without Matrigel (BD Biosciences, USA). A total of 1 × 10^5^ cells were added to the upper inserts. The lower chamber contained 700 μl medium with 20% FBS as a chemoattractant. After incubation for 24–48 h, the cells on the lower surface were fixed with ethanol and stained with 0.2% crystal violet. Relative cell numbers were calculated.

### Wound healing assay

The human colorectal cancer cell line Caco2 cells were examined for their mobility using wound healing assay with ibidi culture insert (Applied Biophysics, USA.) according to the manufacturer’s instructions. Caco2 cells were seeded at a concentration of 5×10^3^/100 μL into individual compartment of DMEM culture insert overnight. The culture plate was filled with DMEM complete medium and the ibidi culture inserts was then removed. A light microscope (DM2000 LED, Leica, Germany) was used to monitor and to take photograph for the migration of the cells once per 24 hours.

### Co-expression network

LncRNA-mRNA co-expression networks were built according to the differentially expressed genes to identify the interactions among genes [11]. Pearson correlation coefficients were calculated to assess the co-expressed relationships between dysregulated lncRNAs and protein-coding mRNAs. The absolute value of correlation coefficients higher than 0.99 with P-value<0.05 was considered statistically significant. The ability of RNAs to interact with other RNAs was quantified using degree and clustering coefficients. The degree represents the individual contribution of one RNA to the other RNAs. The core regulatory factor connected the most adjacent RNAs and had the highest degree. The clustering coefficient represents the density of each gene with the adjacent genes. The greater the clustering coefficient, the more significant the regulatory value of the region in which the gene is located [12]. The co-expression network was established using Cytoscape software.

### Functional enrichment analyses

To evaluate the potential biological processes and pathways that lncRNAs might be involved in, pathway and process enrichment analysis based on the co-expression mRNA was performed using the metascape tool with the following ontology sources: KEGG Functional Sets, GO Cellular Components, GO Molecular Functions, KEGG Pathway, GO Biological Processes, Immunologic Signatures, Oncogenic Signatures, Reactome Gene Sets, Hallmark Gene Sets, Canonical Pathways, Chemical and Genetic Perturbations, BioCarta Gene Sets, CORUM, TRRUST, DisGeNET, PaGenBase, L1000 shRNA, L1000 Compound, L1000 cDNA and L1000 Ligand. Terms with a p-value < 0.01, a minimum count of 3, and an enrichment factor > 1.5 (the ratio between the observed counts and the counts expected by chance) were collected and grouped into clusters. To further capture the relationships between the enriched terms, a subset of enriched terms with the best p-values from each of the 20 clusters were selected to conduct the network and visualized using Cytoscape. The terms with a similarity > 0.3 are connected by edges, and each node represents an enriched term, colored by the cluster ID and the p-value, separately. Hierarchical clustering was used to create the pathway/process clusters of enriched GO terms to find the shared or unique ontology between CRC tumorigenesis and metastasis. In short, after all statistically enriched terms were identified, accumulative hypergeometric p-values and enrichment factors were calculated and used for filtering. The remaining significant terms were then hierarchically clustered into a tree based on the kappa statistical similarities among their gene memberships. Then a 0.3 kappa score was applied as the threshold to cast the tree into term clusters [13].

### Protein-protein interaction network and MCODE algorithm

Protein-protein interaction (PPI) enrichment analysis was carried out using the following databases: BioGrid5, In Web_IM6, OmniPath7 [14]. The Molecular Complex Detection (MCODE) algorithm8 was then applied to identify densely connected network components as a functional description of the corresponding components. The PPI and MCODE algorithms were automatically performed using the metascape tool.

### Bioinformatics Analysis of TCGA-COAD and GSE82236

TCGA-COAD RNA-seq and miRNA data were downloaded from the Genomic Data Commons (GDC) website (https://portal.gdc.cancer.gov/projects/TCGA-COAD). The GSE82236 dataset was downloaded from the Gene Expression Omnibus (GEO) website (https://www.ncbi.nlm.nih.gov/geo/query/acc.cgi?acc=GSE82236). Bioinformatics analyses were completed using the R programming language. The R package library (GDCRNATools), library (edgeR), library (org.Hs.eg.db), library (clusterProfiler), and library (ggplot2) were used to deal with all the data and perform analyses of KEGG functional sets, GO cellular components, GO molecular functions, KEGG pathway, and GO biological processes.

### Statistical analyses

Data are expressed as the mean ± S.D. of three independent experiments and analyzed using the SPSS software program (version 17.0), GraphPad Prism V.9 (GraphPad Prism Software, USA) and statistical programming language R. One-way ANOVA, two-way ANOVA and Wilcoxon rank-sum tests were used for two-group comparisons. The correlation between lncRNA expression and clinicopathologic factors was analyzed using with chi-square test. Cox regression analysis, Kaplan-Meier analysis and the nomogram was constructed using the package of rms and survival in R version. The predictive accuracy of the nomogram was checked by concordance index (C-index). ROC curve was constructed using the package of pROC in R version and the area under the receiver operating curve (ROC) was calculated. Disease-free survival (DFS) duration was defined as the interval from initial surgery to a clinically defined metastasis. Statistical significance was set at P < 0.05.

### Statement of ethics

The study was approved by the ethics committee of The First Affiliated Hospital of Shandong First Medical University.

## RESULTS

### Differential expression analysis of lncRNAs by microarray assay

**Figure 1:**
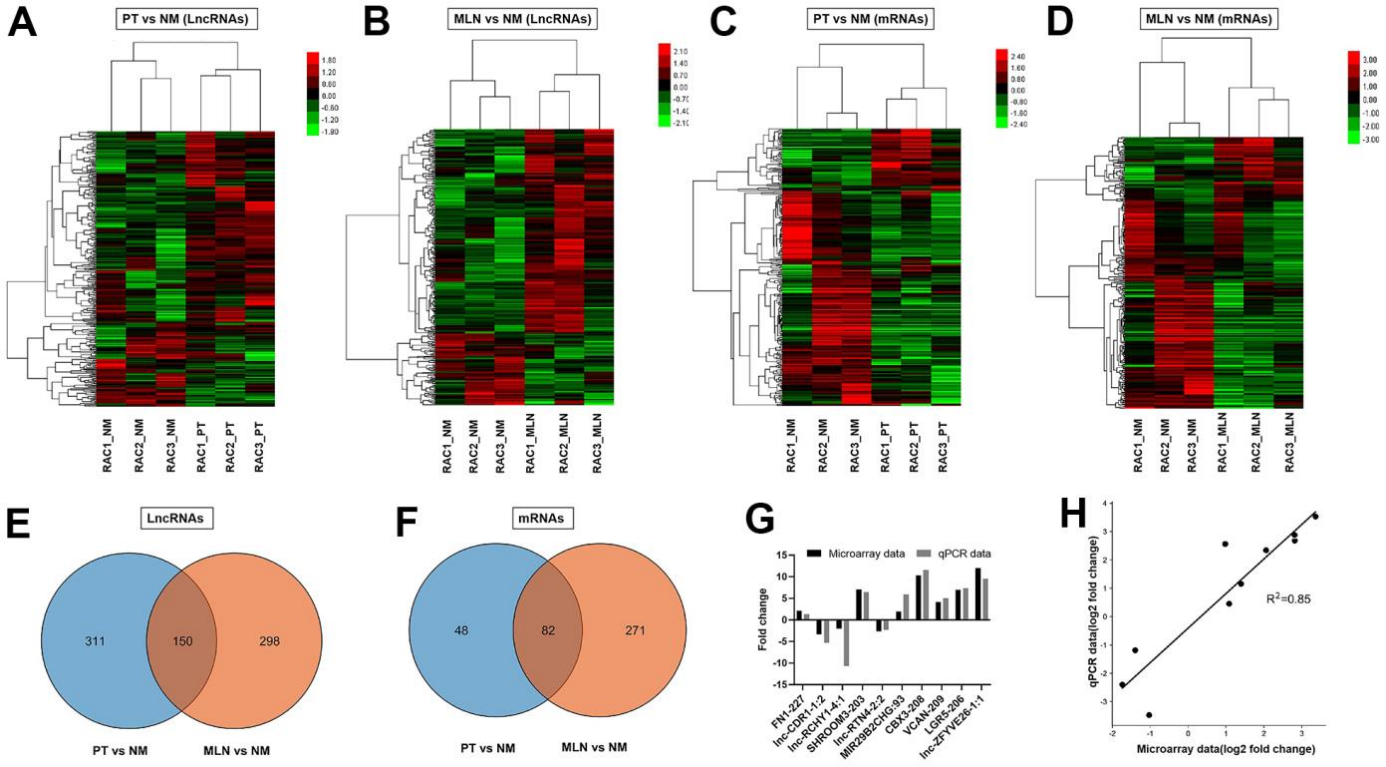
**Differential expression analysis of lncRNAs and mRNAs in CRC.** (**A**) Hierarchical cluster analysis of differentially expressed lncRNAs in primary tumor (PT) versus normal colorectal mucosa tissue (NM). (**B**) Hierarchical cluster analysis of differentially expressed lncRNAs in metastatic lymph nodes (MLNs) versus NM. (**C**) Hierarchical cluster analysis of differentially expressed mRNAs in PT versus NM. (**D**) Hierarchical cluster analysis of differentially expressed mRNAs in MLN versus NM. Venn diagrams showing the numbers of differentially expressed lncRNAs (**E**, **F**) in MLNs compared with those in PTs. (**G**) Verification of microarray data using qRT-PCR (**H**) Correlation analysis of gene expression between microarray data and qRT-PCR data. Microarray data were plotted against data from quantitative real-time PCR. Both the x -and y-axes were shown on a log2 scale. R2 indicates the square of the correlation coefficient.

The microarray data showed that a total of 461 lncRNA transcripts were differentially expressed in the primary tumor (PT) relative to the normal colorectal mucosa (NM), including 331 upregulated transcripts and 130 downregulated transcripts (fold change >1.5, P <0.05). Cluster analysis of differentially expressed lncRNAs in PT versus NM was performed using a heat map (Figure 1A). A total of 448 lncRNA transcripts were differentially expressed in the metastatic lymph node (MLN) relative to NM, including 333 upregulated transcripts and 115 downregulated transcripts (fold change >1.5, P <0.05). Cluster analysis of differentially expressed lncRNAs in MLN versus NM was revealed using a heat map (Figure 1B). The Venn diagram illustrated 150 lncRNAs overlapping between PT and MLN, while most of the differentially expressed lncRNAs were PT-or MLN-specific (Figure 1E). These results indicate a different expression pattern of lncRNAs between PT and MLN. The top 20 dysregulated lncRNAs in PT and MLN were listed in Supplementary Tables 1, 2, respectively.

### Verification of microarray data by qRT-PCR

To validate the reliability of the microarray data, 10 lncRNAs were randomly selected for quantitative real-time PCR (qRT-PCR) analysis. The qRT-PCR results showed a similar expression pattern of lncRNAs as the microarray data (Figure 1G). There was a good correlation (R^2^= 0.85) between the microarray data and qRT-PCR data (Figure 1H). These results indicate that the microarray data could reflect transcriptional dysregulation.

### Differential expression analyses of mRNAs by microarray assay

The microarray data showed that a total of 130 mRNA transcripts were differentially expressed in PT relative to NM, including 70 upregulated transcripts and 60 downregulated transcripts (fold change > 1.5, P < 0.05). Cluster analysis of differentially expressed mRNAs in PT versus NM was revealed using a heat map (Figure 1C). Meanwhile, a total of 353 mRNA transcripts were differentially expressed in MLN relative to NM, including 101 upregulated transcripts and 252 downregulated transcripts (fold change > 1.5, P < 0.05). Cluster analysis of differentially expressed mRNAs in MLN versus NM was revealed using a heat map (Figure 1D). The Venn diagram illustrated that 82 differentially expressed mRNAs overlapped between PT and MLN (Figure 1F).

### Functional analyses of lncRNAs in CRC tumorigenesis

**Figure 2:**
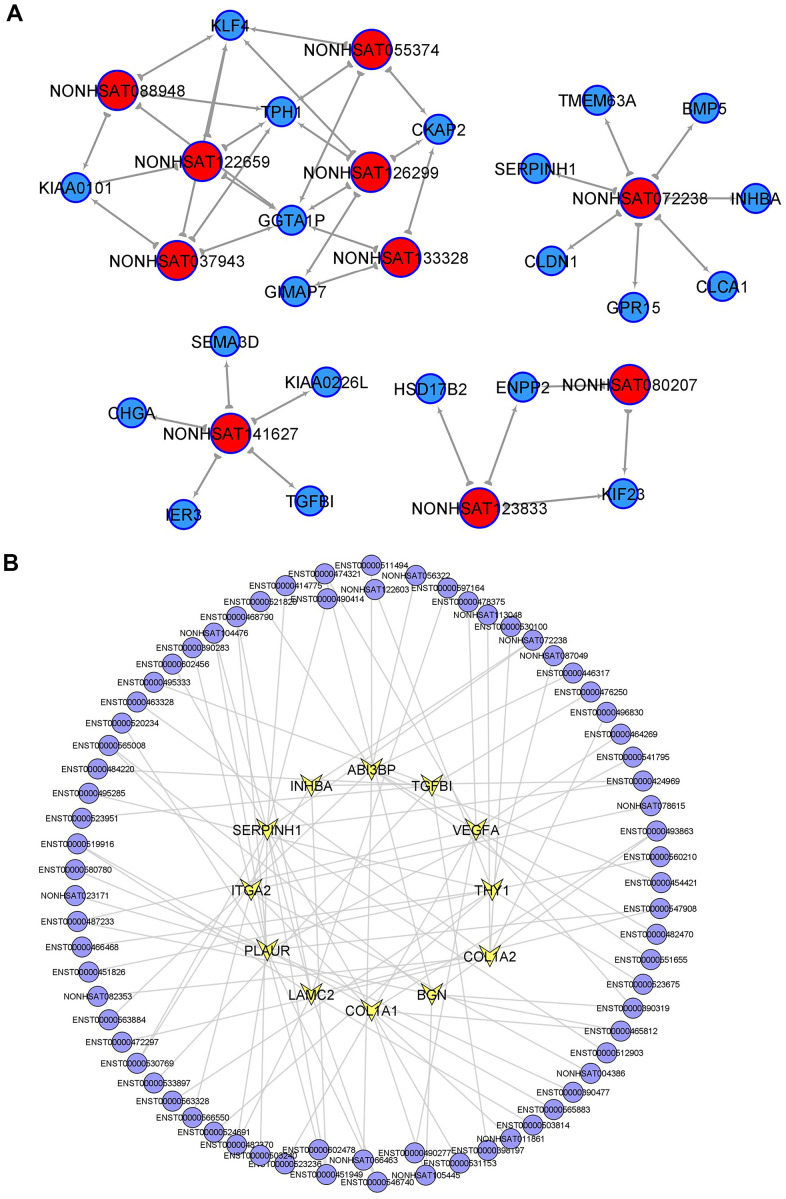
**Key lncRNAs in CRC tumorigenesis and EMT-related lncRNAs.** (**A**) lncRNA-mRNA network in CRC tumorigenesis. Top10 selected lncRNAs and 21 mRNAs were included in this network. (**B**) PT related lncRNAs involved in EMT (epithelial mesenchymal transition) signaling pathway.

To identify the core regulatory transcripts in CRC tumorigenesis, lncRNA-mRNA co-expression networks (Supplementary Figure 1) were constructed based on the correlation between mRNA and lncRNA expression in PT. A total of 383 lncRNAs were with equal to or more than 3 degrees in the lncRNA-mRNA co-expression networks, which were considered as the key lncRNAs in CRC tumorigenesis, and the top 20 core lncRNAs with high degree in PT were listed in Supplementary Table 3. Considering the higher fold change of differential expression in PT compared to NM and higher degree in the lncRNA-mRNA co-expression network in PT, 10 candidate lncRNAs were further selected and considered as the most key CRC tumorigenesis-associated lncRNAs, including (NONHSAT080207 (ZFAS1--TCGA), NONHSAT088948 (LINC01811), NONHSAT122659 (LINC02577), NONHSAT055374 (LINC02563), NONHSAT123833 (LINC-PRSS2-6), NONHSAT072238 (LINC-EIF2AK3-4), NONHSAT141627 (LINC-ZG16-1), NONHSAT126299 (LINC-DKK4-1), NONHSAT133328, NONHSAT037943 (LINC-CIPC-4)]. Each lncRNA and its related mRNA network was constructed (Figure 2A). Moreover, within the 10 tumorigenesis-associated lncRNAs, 7 of them were only found in these samples (LINC02563, lnc-PRSS2-6, lnc-EIF2AK3-4, lnc-ZG16-1, lnc-DKK4-1, NONHSAT133328, and lnc-CIPC-4) compared to TCGA-COAD and GSE82236, and 3 of them (ZFAS1--TCGA, LINC01811, and LINC02577) were found in TCGA-COAD, while no overlapping lncRNAs were found in GSE82236. Therefore, the above 7 lncRNAs may play key roles in CRC tumorigenesis and require further investigation.

**Figure 3:**
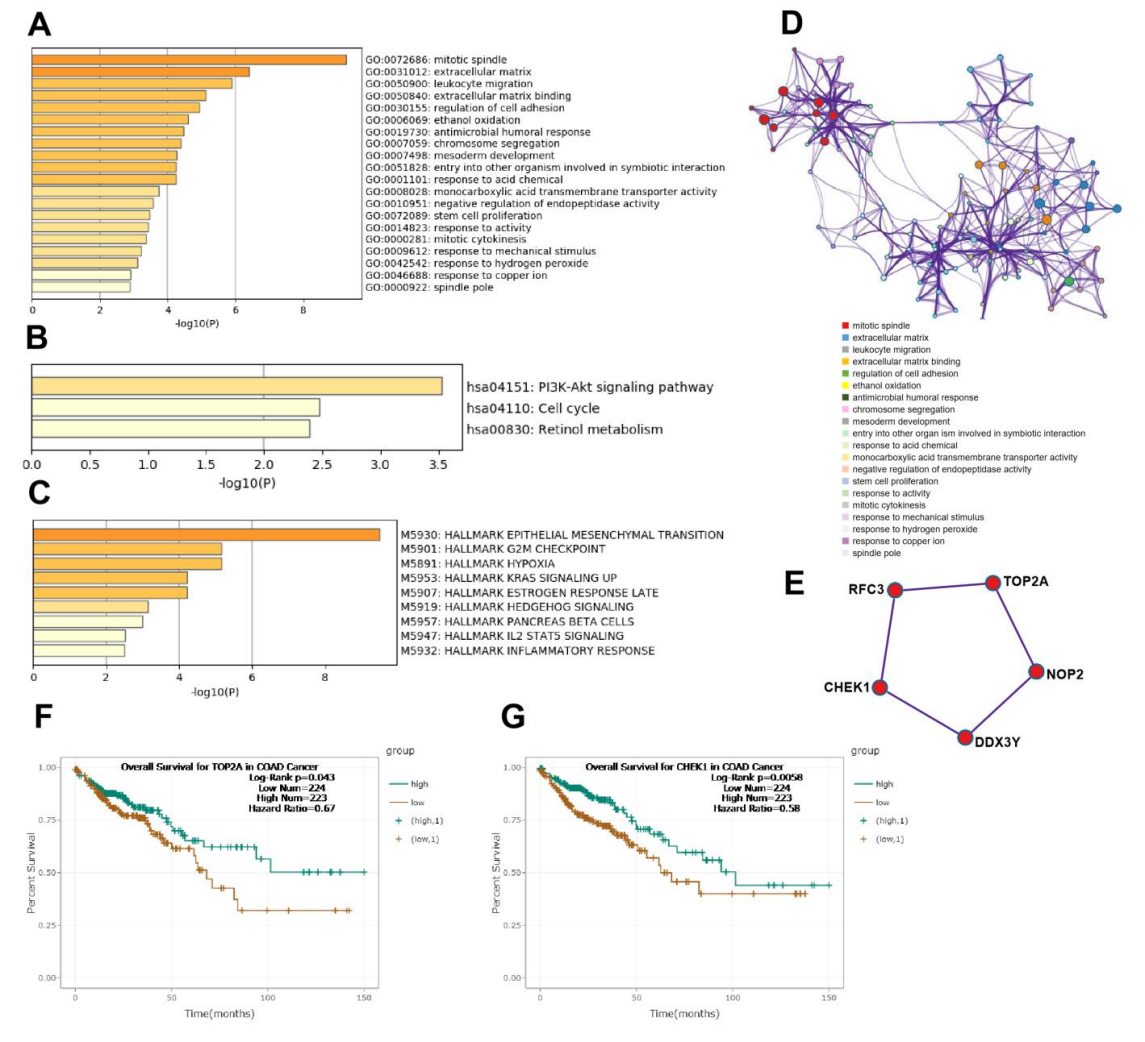
**Functional analysis of lncRNAs in CRC tumorigenesis.** (**A**) GO enrichment showed differentially expressed lncRNA associated biological processes (top 10). (**B**) KEGG pathway analysis showed differentially expressed lncRNAs associated signal pathways (top 10). (**C**) Hallmark Gene Sets analysis showed differentially expressed lncRNAs associated biological processes of diseases and cancer (top 10). (**D**) GO terms network. Each term is represented by a circle node, where its size is proportional to the number of input genes fall into that term, and its color represent its cluster identity. (**E**) The top MCODE network for differentially expressed lncRNAs associated mRNAs. (**F**) Overall survival for patients in TCGA-COAD according to the TOP2A expression level. (**G**) Overall survival for patients in TCGA-COAD according to the CHEK1 expression level.

Subsequently, according to the surrounding 119 mRNAs related to the 383 lncRNAs in the co-expression network, the biological function of lncRNAs in CRC tumorigenesis was predicted through pathway and process enrichment analyses. GO (GO Cellular Components, GO Molecular Functions, GO Biological Processes) analysis showed that the lncRNAs in CRC tumorigenesis were mainly involved in regulating mitotic spindle, extracellular matrix, leukocyte migration, extracellular matrix binding, and regulation of cell adhesion (Figure 3A). Since mitotic spindle misorientation is related to cancer development and progression, lncRNAs may serve as key elements in regulating the assembly of mitotic apparatus to regulate cell growth during CRC tumorigenesis. To further capture the relationships between the enriched terms, the enriched GO term network was rendered. The GO terms network revealed that the function of lncRNAs on the mitotic spindle was more related to chromosome segregation, while the function of lncRNAs on the extracellular matrix (ECM) was more related to cell adhesion (Figure 3D).

Next, KEGG pathway analysis revealed that the lncRNAs in CRC tumorigenesis were primarily involved in the PI3K-Akt signaling pathway, cell cycle, and retinol metabolism (Figure 3B). Furthermore, hallmark gene sets analysis showed that lncRNAs in CRC tumorigenesis were associated with the hallmarks of epithelial mesenchymal, G2M checkpoint, hypoxia, and KRAS signaling (Figure 3C). Protein-protein interaction enrichment analysis and molecular complex detection (MCODE) analysis revealed that five proteins (RFC3, TOP2A, CHEK1, DDX3Y, NOP2) related to P53-signal transduction were identified as the densely connected network components regulated by key lncRNAs in CRC tumorigenesis (Figure 3E). TOP2A and CHEK1 were found to be significantly correlated with the prognosis of patients with TCGA-COAD (Figure 3F, 3G). Lastly, the lncRNAs involved in the regulation of the EMT signaling pathway were further predicted (Figure 2B). Overall, these results showed that lncRNAs in CRC tumorigenesis participated in the regulation of the EMT/P53/PI3K-Akt/KRAS signaling pathway as well as the process related to cell cycle and cell mitosis, which may provide novel clues for the biological effects of lncRNAs on CRC tumorigenesis.

### Functional analyses of mRNAs in CRC tumorigenesis

**Figure 4:**
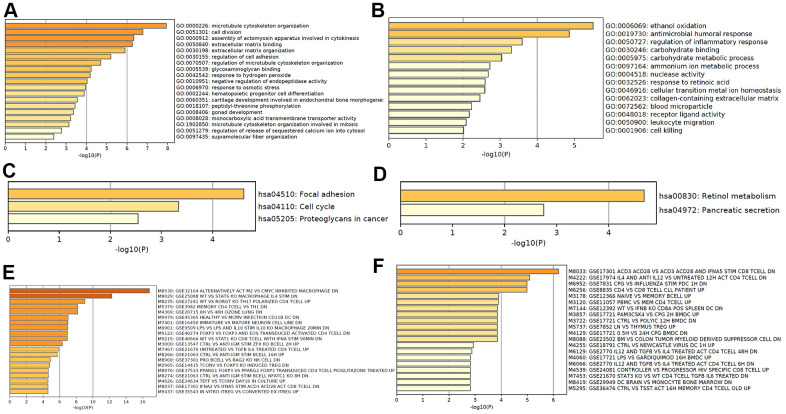
**Functional analysis of mRNA in CRC tumorigenesis.** GO enrichment showed upregulated (**A**) and downregulated (**B**) mRNA in CRC tumorigenesis associated biological processes. KEGG pathway analysis showed upregulated (**C**) and downregulated (**D**) signaling pathways associated with CRC tumorigenesis. Hallmark gene sets analysis showed upregulated (**E**) and downregulated (**F**) expression in CRC tumorigenesis associated immunologic signatures.

Based on the differentially expressed mRNA transcripts in PT relative to NM, pathway and process enrichment analyses were performed to reveal the important biological functions of mRNAs in CRC tumorigenesis. GO analysis showed that 70 upregulated mRNAs in CRC tumorigenesis were mainly involved in regulating microtubule cytoskeleton organization, cell division, assembly of actomyosin apparatus involved in cytokinesis, extracellular matrix binding and organization, and regulation of cell adhesion (Figure 4A), and 60 downregulated mRNAs in CRC tumorigenesis were mainly involved in regulating ethanol oxidation, antimicrobial humoral response, and regulation of inflammatory response (Figure 4B). The KEGG pathway analysis revealed that 70 upregulated mRNAs in CRC tumorigenesis were primarily involved in focal adhesion, cell cycle, and proteoglycans in cancer pathways (Figure 4C), while 60 downregulated mRNAs in CRC tumorigenesis were primarily involved in retinol metabolism and pancreatic secretion pathways (Figure 4D). Immunologic signature analysis was further carried out and showed that the upregulated mRNAs in CRC tumorigenesis were associated with alternatively activated M2 macrophages versus c-MYC inhibited and Th17 polarized CD4 T cells (Figure 4E), while downregulated mRNAs in CRC tumorigenesis were associated with genes downregulated in CD8 T cells activated by CD3/CD28 versus those stimulated by IFNAS, the transcription kinetics initiated by IL-4 in early differentiation of Th2 CD4+T cells, and genes downregulated in plasmacytoid dendritic cells (Figure 4F). Moreover, 119 mRNAs were regarded as core mRNAs with degree>3 in lncRNA-mRNA co-expression networks in CRC tumorigenesis, and the top 20 core mRNAs with high degree in PT were listed in Supplementary Table 4.

### Functional analyses of lncRNAs in CRC metastasis

**Figure 5:**
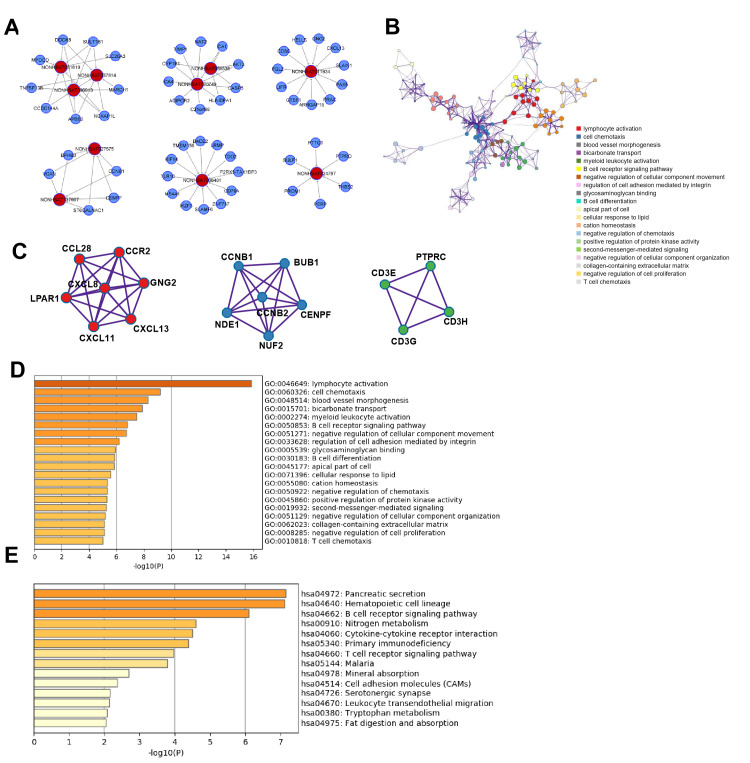
**Functional analysis of lncRNAs in CRC metastasis.** (**A**) lncRNA-mRNA network in CRC metastasis: 10 selected lncRNAs and 53 mRNAs were included in the network. (**B**) GO term network Each term is represented by a circle node, where its size is proportional to the number of input genes falling into that term, and its color represents its cluster identity. (**C**) Top three MCODE networks for differentially expressed lncRNA-associated mRNAs. (**D**) GO enrichment showed differentially expressed lncRNA-associated biological processes (top 10). (**E**) KEGG pathway analysis showed differentially expressed lncRNA-associated signaling pathways (top 10).

Similarly, to identify the core regulatory transcripts in CRC metastasis, lncRNA-mRNA co-expression networks (Supplementary Figure 2) were constructed based on the correlation between mRNA and lncRNA expression in MLN. A total of 295 lncRNAs were with equal to or more than 3 degrees in the lncRNA-mRNA co-expression networks, and the top 20 lncRNAs were listed in Supplementary Table 5, which were considered as the key lncRNAs in CRC metastasis. Considering both higher fold change of differential expression in MLN compared to NM and higher degree in lncRNA-mRNA co-expression network in MLN, 10 candidate lncRNAs were further selected and considered as the most key CRC metastasis-associated lncRNAs, including NONHSAT058538 (LINC-ANKRD30B-2), NONHSAT070749 (LINC-RTN4-2), NONHSAT137607 (FTX), NONHSAT027575 (LINC-AMN1-2), NONHSAT024787 (LINC-BLID-5), NONHSAT037814 (LINC-DLST-2), NONHSAT068481 (LINC-FAM110C-1), NONHSAT096913 (LINC-RCHY1-4), NONHSAT031619 (LINC-DNAH10OS-4), NONHSAT011934 (WAC-AS1). Each lncRNA and its related mRNA network was constructed (Figure 5A). Two of them (FTX and WAC-AS1) have been proved by the TCGA-COAD database to have no significant correlation with the prognosis of colon cancer patients (http://starbase.sysu.edu.cn/panGeneSurvivalExp.php#). However, since the data in the TCGA database were collected only with tumor tissues but not with adjacent normal tissues, the conclusion remains to be further verified. No overlapping LINCRNA was found in GSE82236. Eight of them were first identified and reported by us (LINC-ANKRD30B-2, LINC-RTN4-2, LINC-AMN1-2, LINC-BLID-5, LINC-DLST-2, LINC-FAM110C-1, LINC-RCHY1-4, LINC-DNAH10OS-4), which may play key roles in CRC metastasis and require further investigation.

Based on the 257 mRNAs highly related to the 295 lncRNAs in CRC metastasis, pathway and process enrichment analyses were performed to reveal the important biological functions of lncRNAs in CRC metastasis. GO (GO Cellular Components, GO Molecular Functions, GO Biological Processes) analysis showed that lncRNAs in CRC metastasis were mainly involved in lymphocyte activation, cell chemotaxis, blood vessel morphogenesis, bicarbonate transport, myeloid leukocyte activation, and B cell receptor signaling pathway (Figure 5D). The enriched GO terms network showed that there was a strong correlation among lymphocyte activation, myeloid leukocyte activation, and B cell receptor signaling pathway, suggesting that lncRNAs in CRC metastasis played an important role in tumor immunity, and cell chemotaxis located at the core of the network and blood vessel morphogenesis was more related to negative regulation of cellular component movement. (Figure 5B).

KEGG pathway analysis revealed that lncRNAs in CRC metastasis mainly participated in the regulation of pancreatic secretion, hematopoietic cell lineage, B cell receptor signaling pathway, nitrogen metabolism, cytokine-cytokine receptor interaction, and T cell receptor signaling pathway(Figure 5E).

Next, protein-protein interaction enrichment analysis and the MCODE analysis showed that three MCODEs were identified as densely connected network components, including chemokine receptor binding, condensed chromosome kinetochore, positive thymic T cell selection, and T cell receptor signaling pathway (Figure 5C). Furthermore, the lncRNAs involved in lymphocyte activation were further predicted (Supplementary Figure 3A, 3B).

In summary, lncRNAs in CRC metastasis predominantly regulated immune and inflammation-related biological processes, including immune cell activation, cell chemotaxis, cytokine signaling pathways, and blood vessel morphogenesis, suggesting that lncRNAs may control early lymph node metastasis of CRC by regulating tumor immunity and blood vessel morphogenesis.

### Functional analyses of mRNAs in CRC metastasis

**Figure 6:**
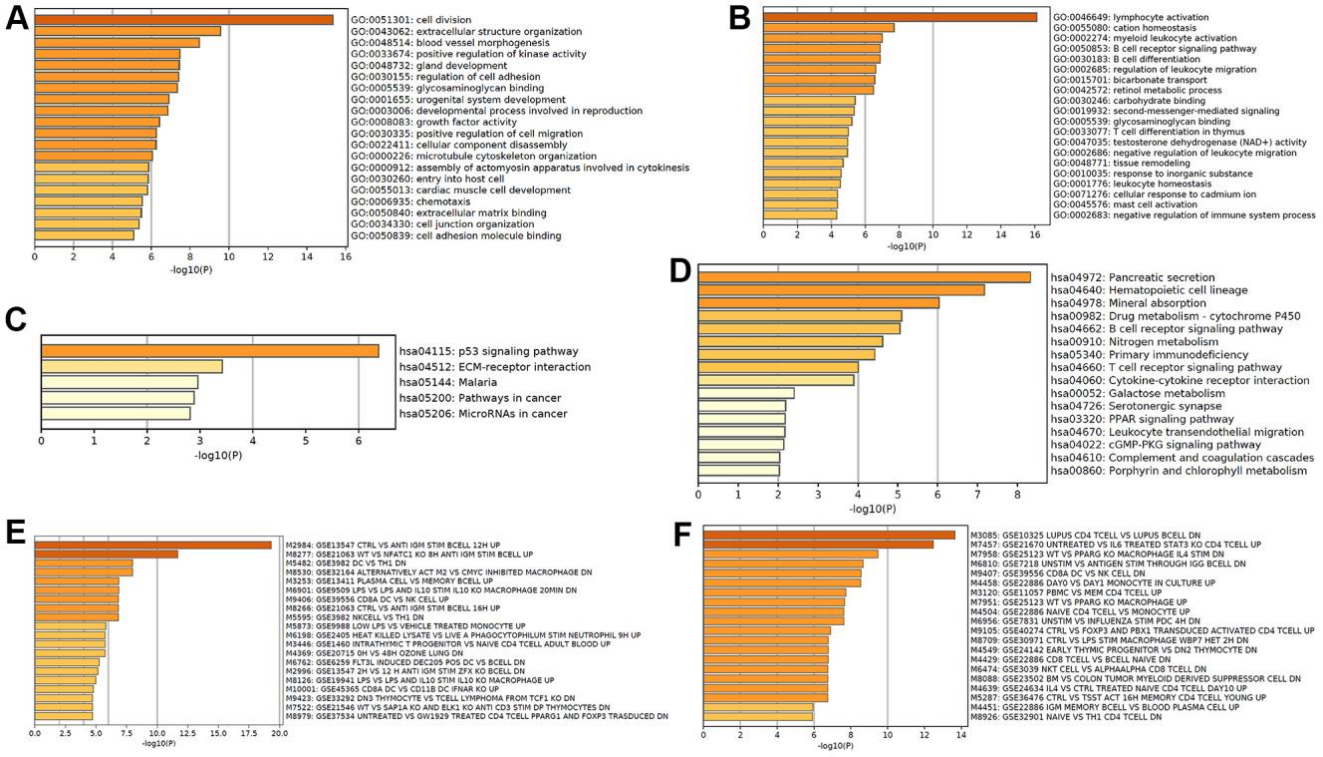
**Functional analysis of mRNA in CRC metastasis.** GO enrichment showed upregulated (**A**) and downregulated (**B**) mRNAs in CRC metastasis-associated biological processes. KEGG pathway analysis showed upregulated (**C**) and downregulated (**D**) CRC metastasis-associated signaling pathways. Hallmark gene sets analysis showed upregulated (**E**) and downregulated (**F**) expression in CRC metastasis-associated immunologic signatures.

Based on the differentially expressed mRNA transcripts in MLN relative to NM, pathway and process enrichment analyses were performed to reveal the important biological functions of mRNAs in CRC metastasis. GO analysis showed that 101 upregulated mRNAs in CRC metastasis were mainly involved in regulating cell division and extracellular structure organization, which were similar to GO in CRC tumorigenesis, as well as blood vessel morphogenesis, which may facilitate cancer metastasis (Figure 6A), while 252 downregulated mRNAs in CRC metastasis were mainly involved in regulating lymphocyte activation, cation homeostasis, myeloid leukocyte activation, BCR signaling pathway, B cell differentiation, and leukocyte migration, suggesting that cancer metastasis was related to tumor immune escape (Figure 6B). The KEGG pathway analysis revealed that upregulated mRNAs in CRC metastasis were primarily involved in the p53 signaling pathway, ECM-receptor interaction, and pathways in cancer (Figure 6C), while downregulated mRNAs in CRC metastasis were primarily involved in pancreatic secretion pathways, which were similar to those in tumorigenesis, hematopoietic cell lineage, mineral absorption, drug metabolism, and B cell receptor signaling pathway (Figure 6D). Immunologic signature analysis was further carried out and showed that the upregulated mRNAs in CRC metastasis were associated with genes upregulated in B lymphocytes and downregulated genes in comparison with DC versus Th1 cells (Figure 6E), while downregulated mRNAs in CRC metastasis were associated with genes regulated in CD4 T cells (Figure 6F). Moreover, 257 mRNAs were regarded as core mRNAs with degree>3 in lncRNA-mRNA co-expression networks in CRC metastasis and the top 20 core mRNAs with high degree in MLN were listed in Supplementary Table 6.

### Functional comparison of lncRNAs: CRC tumorigenesis versus CRC metastasis

**Figure 7:**
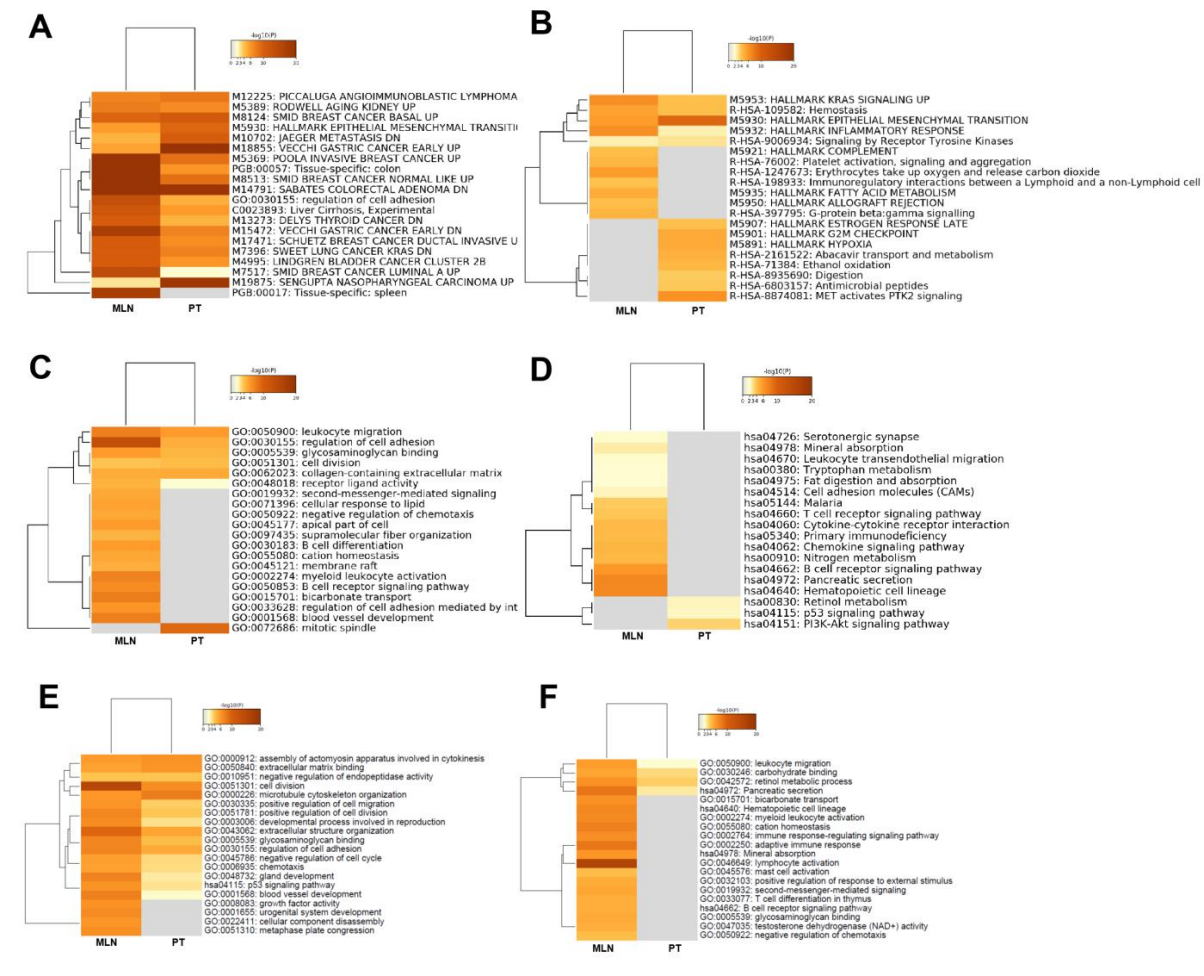
**Functional comparison of lncRNAs and mRNAs: CRC tumorigenesis versus CRC metastasis.** Functional comparison analysis of lncRNAs: (**A**) Heatmap cluster analysis of enriched ontology terms (top 10). (**B**) Heatmap cluster analysis of reactome gene sets and hallmark gene set enrichment (top 10). (**C**) Heatmap cluster analysis of GO enrichment (top 10). (**D**) Heatmap cluster analysis of KEGG pathway (top 10). Functional comparison of upregulated (**E**) and downregulated (**F**) mRNAs in CRC tumorigenesis and metastasis using heatmap cluster analysis of enriched ontology terms and KEGG pathway.

To directly compare the differences in biological functions of lncRNAs in CRC tumorigenesis and metastasis, heatmap cluster analysis of enriched terms was performed utilizing the following ontology sources: KEGG functional sets, GO cellular components, GO molecular functions, KEGG pathway, GO biological processes, immunological signatures, oncogenic signatures, reactome gene sets, hallmark gene sets, canonical pathways, chemical and genetic perturbations, BioCarta gene sets, CORUM, TRRUST, DisGeNET, PaGenBase, L1000 shRNA, L1000 Compound, L1000 cDNA, and L1000 Ligand. The results showed that the term Sabates colorectal adenoma DN was enriched both in CRC tumorigenesis and in CRC metastasis, suggesting that lncRNAs had a regulatory role in the colorectal adenoma-carcinoma transition (Figure 7A). The specific gene sets and functional sets enrichment analysis indicated that lncRNAs in CRC tumorigenesis predominantly regulated tumorigenesis-related biological processes, such as the P53 signaling pathway, KRAS signaling pathway, MET activates the PTK2 signaling pathway, cell cycle, cell growth, and cell metabolism as well as the process of EMT, while lncRNA CRC metastasis predominantly regulated immunity-related biological processes as well as blood vessel morphogenesis, which contributes to cancer metastasis (Figure 7B–7D).

In summary, lncRNAs regulated diverse signaling pathways and biological processes in CRC tumorigenesis and metastasis, which may lead to a better understanding of the biological functions of lncRNAs in the progression of CRC.

### Functional comparison of mRNAs: CRC tumorigenesis versus CRC metastasis

To directly compare the differences in biological functions involved by mRNAs between CRC tumorigenesis and metastasis, heatmap cluster analysis of enriched terms was performed using the following ontology sources: KEGG functional sets, GO cellular components, GO molecular functions, KEGG pathway, and GO biological processes. The results showed that cell division and extracellular structure organization were enriched in upregulated mRNAs in both CRC tumorigenesis and CRC metastasis (Figure 7E), while GO and signaling pathways of growth factor activity, urogenital system development, cellular component disassembly, and metaphase plate congression were deleted in CRC tumorigenesis. For downregulated mRNAs, the tumor immune responses, such as lymphocyte activation and adaptive immune response, were enriched in CRC metastasis, but were deleted in CRC tumorigenesis (Figure 7F).

### LncRNA MIR29B2CHG93 promoted cell proliferation and tumor metastasis in CRC

**Figure 8:**
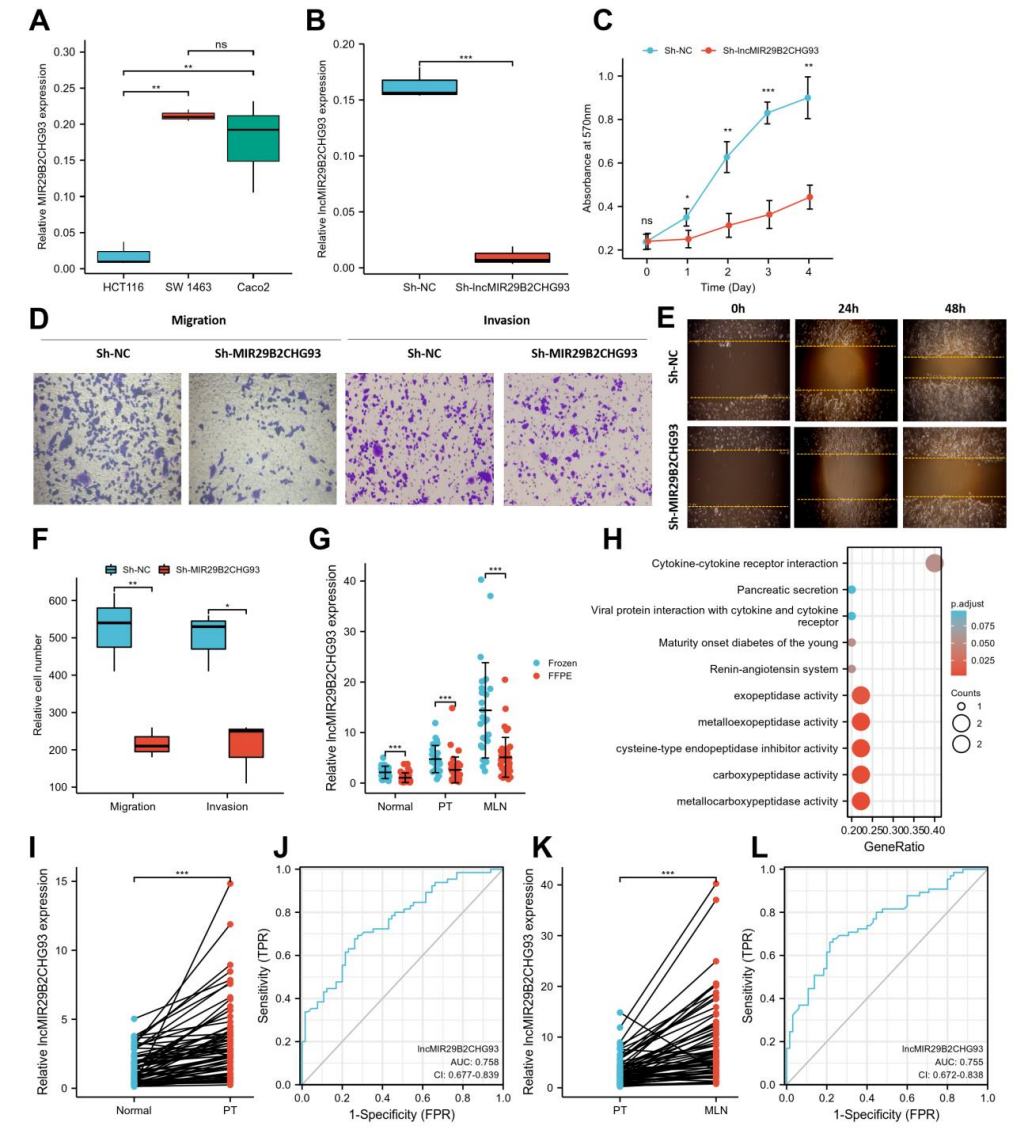
**LncRNA MIR29B2CHG93 promoted cell proliferation and tumor metastasis in CRC.** (**A**) RT-PCR analysis was used to detect the expression of lncRNA MIR29B2CHG93 in cell lines. (**B**) The expression levels of lncRNA MIR29B2CHG93 in Caco2 cells after transfection with sh-NC or sh-MIR29B2CHG93 were detected by RT-PCR. (**C**) The effects of lncRNA MIR29B2CHG93 knockdown on the proliferation of Caco2 cells were examined by MTT assay. (**D**–**F**) Transwell assay and wound healing assay were used to evaluate the migration and invasion ability of Caco2 cells transfected with sh-NC or sh-MIR29B2CHG93. (**D**) Images of Caco2 cells in migration and invasion transwell assays. (**E**) Cell mobility was determined by wound healing assay at 0, 24, 48h after the scratching. (**F**) Quantification of cell migration and invasion in (**D**). (**G**) The expression of lncRNA MIR29B2CHG93 was compared between in frozen tissue and FFPE tissues in normal mucosa, primary tumor and lymphnodal metastasis tumor tissue. (**H**) GO and KEGG analysis of lncRNA MIR29B2CHG93 based on co-expressed mRNAs. (**I**) LncRNA MIR29B2CHG93 expression in primary tumors compared with paired normal tissues from CRC cohort. (**J**) A ROC curve for assessing the predictive ability of lncRNA MIR29B2CHG in predicting normal and tumor. (**K**) LncRNA MIR29B2CHG93 expression in lymphnodal metastasis tumors compared with paired primary tumors from CRC cohort. (**L**) A ROC curve for assessing the predictive ability of lncRNA MIR29B2CHG in predicting lymphnodal metastasis. *P<0.05, **P<0.01, ***P<0.001.

In the present study, we mainly focused on the upregulated lncRNAs, given that these lncRNAs might serve as therapeutic targets or prognostic biomarkers. Among them, lncRNA MIR29B2CHG93, which was one of the most prominently upregulated lncRNAs, was chosen for further evaluation. LncRNA MIR29B2CHG93, a lincRNA and antisense lncRNA gene, is located on 1q32.2 in humans and is composed of one exon with a full length of 2559 bp. The expression of lncRNA MIR29B2CHG93 in three CRC cell lines showed that lncRNA MIR29B2CHG93 was significantly higher in Caco2 and SW 1463 cells than in HCT116 cells (Figure 8A). Therefore, Caco2 cells, which were highly differentiated colon cancer cells that express wild-type KRAS, were chosen for further assays. To determine the biological function of lncRNA MIR29B2CHG93 in CRC cells, lncRNA MIR29B2CHG93 expression was silenced using a lentivirus-mediated short hairpin RNA (shRNA) approach in the CRC cell line Caco2, and the knockdown efficiency was confirmed by RT-PCR (Figure 8B). After lncRNA MIR29B2CHG931 knockdown, the proliferation of CRC cells was significantly decreased (Figure 8C). To investigate the role of lncRNA MIR29B2CHG93 in the migration and invasion ability of CRC cells, we performed transwell migration and invasion assay (Figure 8D, 8F) as well as wound healing assay (Figure 8E). These data showed that lncRNA MIR29B2CHG93 significantly impaired the migration and invasion of CRC cells.

To elucidate the association of lncRNA MIR29B2CHG93 with the clinicopathological characteristics of CRC, we further investigated the expression of lncRNA MIR29B2CHG93 in paired CRC tissues, including normal mucosa, primary tumor, and lymph nodal metastasis tumor tissue from 65 CRC patients using real-time PCR analysis. The clinicopathological characteristics of CRC patient cohort were summarized in Table 1. The lncRNA MIR29B2CHG93 was significantly overexpressed in frozen tissues (n=25) compared with FFPE tissues (n=40), regardless of the normal mucosa, primary tumor, and lymph nodal metastasis tumor tissue (Figure 8G). Furthermore, the lncRNA MIR29B2CHG93 was significantly upregulated in CRC primary tumor tissues compared with adjacent normal tissues (Figure 8I), while the lncRNA MIR29B2CHG93 was upregulated in CRC cancer tissues with lymph node metastasis compared with that in paired CRC primary tumor tissues(Figure 8K). The correlation between lncRNA MIR29B2CHG93 expression in PT or MLN and clinicopathologic factors, including age, gender, nodal stage, tumor site and histological grade, were analyzed and the result showed no significance, except for the tumor site (Supplementary Figure 4). ROC curve analysis validated that lncMIR29B2CHG93 had certain predictive ability in distinguishing between normal and tumor (AUC = 0.758, CI = 0.677-0.839, Figure 8J), and whether there was lymph node metastasis (AUC = 0.755, CI = 0.672-0.838, Figure 8L). To predict the biological function of lncMIR29B2CHG93, GO and KEGG analyses were performed based on the co-expressed mRNAs and showed that lncMIR29B2CHG93 might be involved in the biological process of metallocarboxypeptidase activity, carboxypeptidase activity, cysteine-type endopeptidase inhibitor activity, metalloexopeptidase activity, exopeptidase activity and cytokine-cytokine receptor interaction signaling pathway (Figure 8H).

**Table 1:** Clinical pathological characteristics of CRC patients.

**Characteristic**	**Levels**	**Overall**
n		65
Gender, n (%)	Female	26 (40%)
	Male	39 (60%)
T stage, n (%)	T2	2 (3.1%)
	T3	41 (63.1%)
	T4	22 (33.8%)
N stage, n (%)	N1	39 (60%)
	N2	26 (40%)
M stage, n (%)	M0	63 (96.9%)
	M1	2 (3.1%)
AJCC stage, n (%)	IIA	2 (3.1%)
	IIIA	1 (1.5%)
	IIIB	44 (67.7%)
	IIIC	16 (24.6%)
	IV	2 (3.1%)
Histological grade, n (%)	Grade 2	43 (66.2%)
	Grade 3	22 (33.8%)
Histological classification, n (%)	Mucinous adenocarcinoma	2 (3.1%)
	Nonmucinous adenocarcinoma	63 (96.9%)
Tumor site, n (%)	Colon	39 (60%)
	Rectum	26 (40%)
Age, median (IQR)		62 (52, 70)

### High expression of lncRNA MIR29B2CHG was indicator of unfavorable clinical outcome in CRC

**Figure 9:**
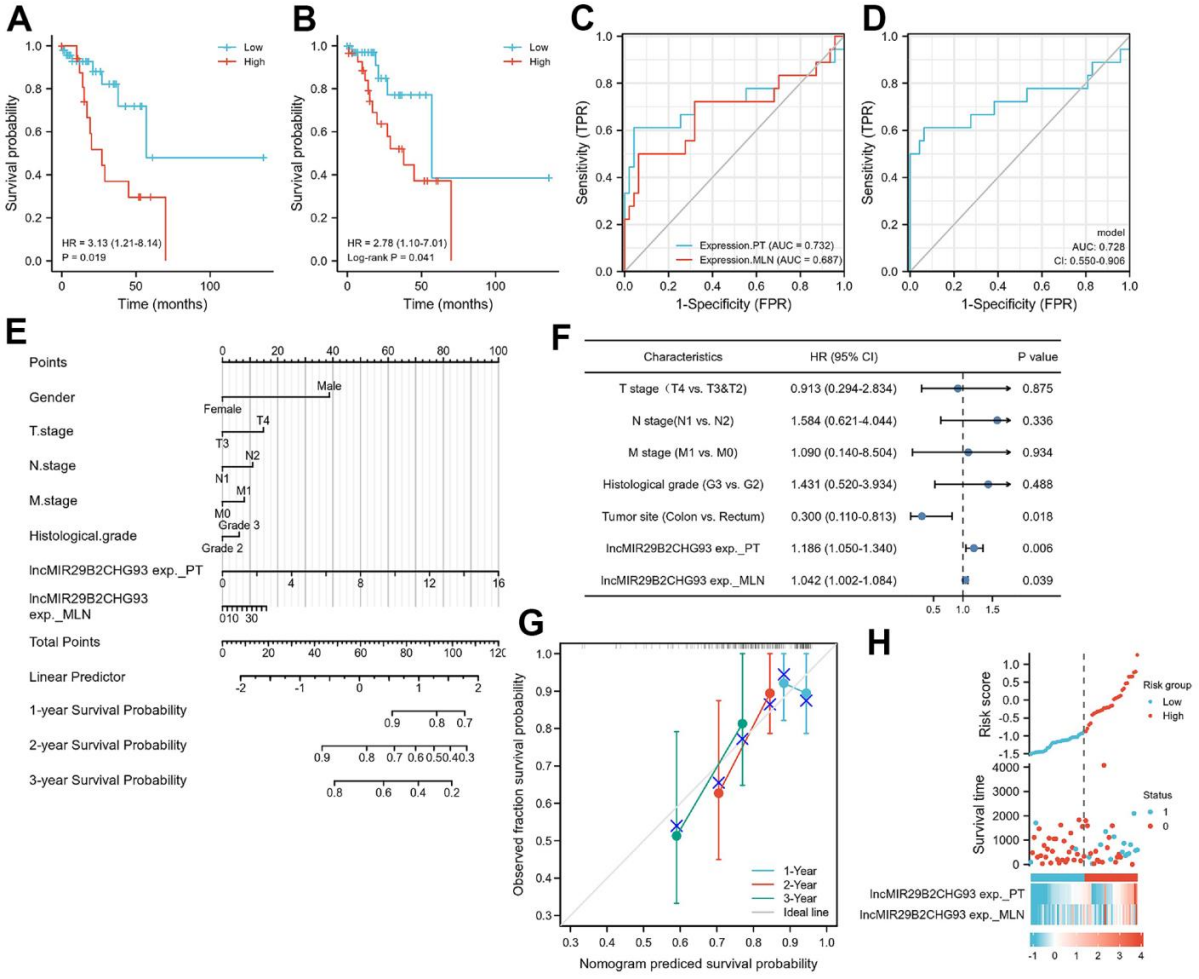
**Prognostic model based on lncRNA MIR29B2CHG expression in CRC patients.** Kaplan–Meier analysis of Disease-free survival(DFS) of CRC patients based on lncRNA MIR29B2CHG expression in primary tumor tissue (**A**) and in lymphnodal metastasis tumor tissue (**B**). Data are shown as hazard ratios (95% CI). (**C**) Time-dependent ROC curve for assessing the prognostic accuracy of CRC patients by the lncRNA MIR29B2CHG expression in primary tumor and lymphnodal metastasis tumor tissue, separately. (**D**) Time-dependent ROC curve for assessing the prognostic accuracy of CRC patients by the joint expression of lncRNA MIR29B2CHG in both primary tumor and lymphnodal metastasis tumor tissue. Data are shown as AUC (95% CI). ROC = receiver operator characteristic. AUC = area under the curve. (**E**) The nomogram was utilized by adding up of the points identified on the points scale for each variant. The total points occurred on the bottom scales represent the probability of 1-, 2- and 3-year survival. (**F**) Univariate analysis was performed in CRC cohort. The bar corresponds to 95% confidence intervals. (**G**) The calibration curve based on the expression of lncRNA MIR29B2CHG in primary tumor and lymphnodal metastasis tumor tissue for predicting DFS at 1-, 2- and 3-years in CRC cohort. (**H**) The distribution of risk score of the established prognostic model by lncRNA MIR29B2CHG expression.

Kaplan-Meier analysis showed that high expression of lncRNA MIR29B2CHG in primary tumor tissues were strongly associated with poorer DFS (HR=3.13, P=0.019, Figure 9A), as well as in lymph nodal metastasis tumor tissue (HR=2.78, log-rank P = 0.041, Figure 9B). A Cox proportional hazards model was used to build a prognostic classifier. Based on this lncRNA MIR29B2CHG expression, we assessed the prognostic accuracy of the risk score with a time-dependent ROC analysis, it trended towards a higher prognostic accuracy in PT than in MLN (AUC for PT=0.732, CI = 0.556-0.907; AUC for MLN=0.687, CI=0.515-0.859; Figure 9C). DeLong’s test showed that lncRNA MIR29B2CHG expression in PT was better than lncRNA MIR29B2CHG expression in MLN in predicting the DFS status, whereas the results were not statistically significant (P=0.470). When combining the expression of lncRNA MIR29B2CHG in both PT and MLN with a time dependent ROC analysis, it still trended towards high prognostic accuracy (AUC=0.728; CI=0.550–0.906; Figure 9D). To further optimize this classifier, some pathological variables and lncRNA MIR29B2CHG expression were subjected to a univariate Cox analysis. The result revealed that lncRNA MIR29B2CHG high expression correlated significantly with a poor DFS (PT: HR=1.186; CI=1.050-1.340; P =0.006; MLN: HR=1.042; CI=1.002-1.084; P =0.039, Figure 9F). Using the multivariate Cox proportional hazards model, a prognostic nomogram was established based on selected risk factors with high hazard ratios to predict the probability of recurrence-free survival through the scores identified on the points scale for every risk factor and the C-index for DFS prediction of the formulated nomogram was 0.644 (95% CI: 0.558-0.730; P=0.111, Figure 9E). The calibration plot for the probability of recurrence-free survival based on the expression of lncRNA MIR29B2CHG fitted well between the prediction by nomogram and actual observation (Figure 9G). For further assess the prognostic performance of lncRNA MIR29B2CHG, the patients were divided into a high-risk group and low risk group according to their median risk score and lncRNA MIR29B2CHG inclined to be highly expressed in patients with high-risk scores (Figure 9H). Collectively, these findings revealed that high expression of lncRNA MIR29B2CHG was indicator of unfavorable clinical outcome in CRC.

## DISCUSSION

With the development of sequencing and bioinformatics analysis technology [15, 16], an increasing amount of tumor-related information has been discovered. In view of the flaws of TCGA-COAD data and the advances in CRC [1, 9], this study was designed and put into practice. Analysis of the chip sequencing results revealed that the expression of lncRNA in PT or MLN was different from that in NM (Figure 1A, 1B). When PT was compared with MLN, it was found that the lncRNAs involved in the two were also significantly different (Figure 1E). Therefore, we inferred that this may be due to the participation of a large number of new lncRNAs that led to the occurrence of MLN. Furthermore, the consistency between the chip sequencing and Q-PCR results further supported our inference (Figure 1G, 1H).

A similar trend was observed when mRNAs were analyzed (Figure 1C, 1D, 1F). As previously reported, the occurrence and development of cancer is a complex process involving multiple steps, stages, and genes [17]. Therefore, we infer that the emergence of new genes and lncRNAs may be important contributors to disease progression. If the function of these abnormal genomic alterations and the specific mechanism of action can be clarified, it may be of great help in future anti-tumor treatments. Therefore, their functional analyses were put into practice (Figures 2–7).

The correlation network between lncRNAs and mRNAs was shown in (Supplementary Figure 1). Within the top10 tumorigenesis-associated lncRNAs, 7 were found only in these samples compared to TCGA-COAD and GSE82236, and 3 of them were found in TCGA-COAD, while no overlapping lncRNAs were found in GSE82236. Therefore, similar to a previous report [7, 18], we concluded that 7 unique lncRNAs may play key roles in CRC tumorigenesis, and further investigations are needed.

According to the surrounding 119 mRNAs related to the 383 lncRNAs in the co-expression network, the biological function of lncRNA in CRC tumorigenesis was predicted through pathway and process enrichment analyses. Mitotic spindles, rarely reported in colorectal cancer but for breast cancer [19], was found to be at the forefront of the enrichment results (Figure 3A). Since mitotic spindle misorientation is related to cancer development and progression [19], lncRNAs may serve as key elements in regulating the assembly of mitotic apparatus to regulate cell growth during CRC tumorigenesis (Figure 3A). Similar results were also found in mRNA analyses related to microtubule cytoskeleton organization (Figure 4A). These results suggest that in the process of CRC tumorigenesis, new lncRNA and mRNA genes are involved in driving this event (Figures 3A, 4A, 4B). When lncRNAs involved in KEGG pathway analysis were carried out, these lncRNAs were first discovered in the classic PI3K-Akt signaling pathway related to cancer (Figure 3B) [20]. Therefore, we speculate that these lncRNAs regulate CRC tumorigenesis mainly through the PI3K-Akt signaling pathway, cell cycle, and retinol metabolism (Figure 3B).

Further analysis of hallmark gene sets showed that these lncRNAs in CRC tumorigenesis were also associated with hallmarks of epithelial mesenchymal, G2M checkpoint, hypoxia, and KRAS signaling (Figure 3C), which only KRAS was often mentioned in colorectal cancer [21, 22]. Five proteins (RFC3, TOP2A, CHEK1, DDX3Y, and NOP2) related to P53-signal transduction were identified as the densely connected network components regulated by key lncRNAs in CRC tumorigenesis (Figure 3E). TOP2A and CHEK1 were found to be significantly correlated with the prognosis of patients with TCGA-COAD (Figure 3F, 3G), indicating that both of them had the potential to be biomarkers for the prognosis of CRC. The lncRNAs involved in the regulation of the EMT signaling pathway were further predicted (Figure 2A, 2B). The results showed that lncRNAs in CRC tumorigenesis participated in the regulation of the EMT/P53/PI3K-Akt/KRAS signaling pathway as well as the processes related to cell cycle and cell mitosis [23, 24], which may provide novel clues for the biological effects of lncRNAs on CRC tumorigenesis [25].

Similar functional analyses were also used for CRC metastasis, and relevant abnormal lncRNAs and mRNAs were also found (Figures 5, 6, 7). Whether it is for differentially expressed lncRNAs (Figure 5) or mRNAs (Figure 6), or the results of enrichment analyses, it is suggested that the related lncRNAs and mRNAs involved in CRC tumorigenesis and metastasis are different, which was verified by our subsequent analysis (Figure 7). In summary, differentially expressed lncRNAs and mRNAs regulated diverse signaling pathways and biological processes in CRC tumorigenesis and metastasis, which may lead to a better understanding of the biological functions of lncRNAs and mRNAs in the progression of CRC.

Early and accurate prediction of CRC patients with metastatic lesions could help in more effective prevention and treatment. It is of great importance to comprehensively understand the molecular mechanisms involved in CRC metastasis. LncRNAs have been shown to play important roles in tumor progression, and many studies have focused on the functions and regulation of lncRNAs to identify novel molecular targets for the diagnosis and treatment of CRC. In this study, Functional studies revealed that the uncharacterized lncRNA MIR29B2CHG93 could promote the proliferation and mobility of CRC cells *in vitro* (Figure 8C–8F), indicating a tumor-promoter role of lncRNA MIR29B2CHG93 in CRC. We found that the potential target genes regulated by lncMIR29B2CHG93 were enriched in the biological process of metallocarboxypeptidase activity, carboxypeptidase activity, cysteine-type endopeptidase inhibitor activity, metalloexopeptidase activity, exopeptidase activity and cytokine-cytokine receptor interaction signaling pathway (Figure 8H). Therefore, it is necessary to further study to elucidate the more specific molecular mechanism triggered by lncMIR29B2CHG93, which is helpful for the development of new drug candidates for targeted therapy of CRC.

LncRNA MIR29B2CHG93 was significantly increased in CRC primary tumor tissues compared to normal tissues (Figure 8I), and ROC curve analysis validated that lncMIR29B2CHG93 had certain predictive ability in distinguishing between normal and tumor (AUC = 0.758, CI = 0.677-0.839, Figure 8J), suggesting that lncRNA MIR29B2CHG93 may serve as a diagnostic biomarker for CRC. Lymph node metastases are thought to occur before distant metastasis. Furthermore, lncRNA MIR29B2CHG93 was upregulated in metastatic lymph node tissue compared with that in paired CRC primary tumor tissues (Figure 8K) and ROC curve analysis validated that lncMIR29B2CHG93 had certain predictive ability in distinguishing whether there was lymph node metastasis (AUC = 0.755, CI = 0.672-0.838, Figure 8L), suggesting that lncRNA MIR29B2CHG93 may serve as an early predictor of metastasis.

Noteworthy, patients with lymph node metastasis and surgical indications were selected in this study and the AJCC staging and tumor staging among patients were similar, which may lead to that there were no significant correlation between lncRNA MIR29B2CHG93 expression and clinicopathologic factors (Supplementary Figure 4). But importantly, we found in the Kaplan–Meier survival analysis that patients with high lncRNA MIR29B2CHG93 expression showed poor DFS than those with low lncRNA MIR29B2CHG93 expression (Figure 9A, 9B). Furthermore, we found that lncRNA MIR29B2CHG93 trended towards a high prognostic accuracy (Figure 9C, 9D) and lncRNA MIR29B2CHG93 expression was poor prognostic factors for CRC (Figure 9E–9H). Altogether, these results demonstrated that lncRNA MIR29B2CHG93 can be used as biomarkers for diagnosis, prognosis and metastasis-prediction in CRC patients.

## CONCLUSIONS

In summary, this study revealed different expression patterns and biological functions of lncRNAs and mRNAs between CRC tumorigenesis and metastasis, as well as novel tumorigenesis-associated lncRNAs and metastasis-associated lncRNAs, which provided new insights for an in-depth understanding of the mechanism of tumorigenesis and metastasis of CRC. Moreover, we identified lncRNA MIR29B2CHG93 as a tumor promoter in CRC, and the higher expression of lncRNA MIR29B2CHG93 was indicator of unfavorable clinical outcome in CRC. Our results provide a better understanding of the role of lncRNAs in CRC progression and a potential therapeutic target and prognostic predictor of this malignancy.

## Supplementary Material

Supplementary Figures

Supplementary Tables
