# Supplementary material for: Identification and functional analysis of lncRNAs and mRNAs between tumorigenesis and metastasis in CRC
Source: Aging (Albany NY). 2021 Dec 26;13(24):25859–85. doi: 10.18632/aging.203775 (PMC8751602; doi:10.18632/aging.203775)
Supplement: Supplementary Figures [file aging-13-203775-s001.pdf]

SUPPLEMENTARY FIGURES

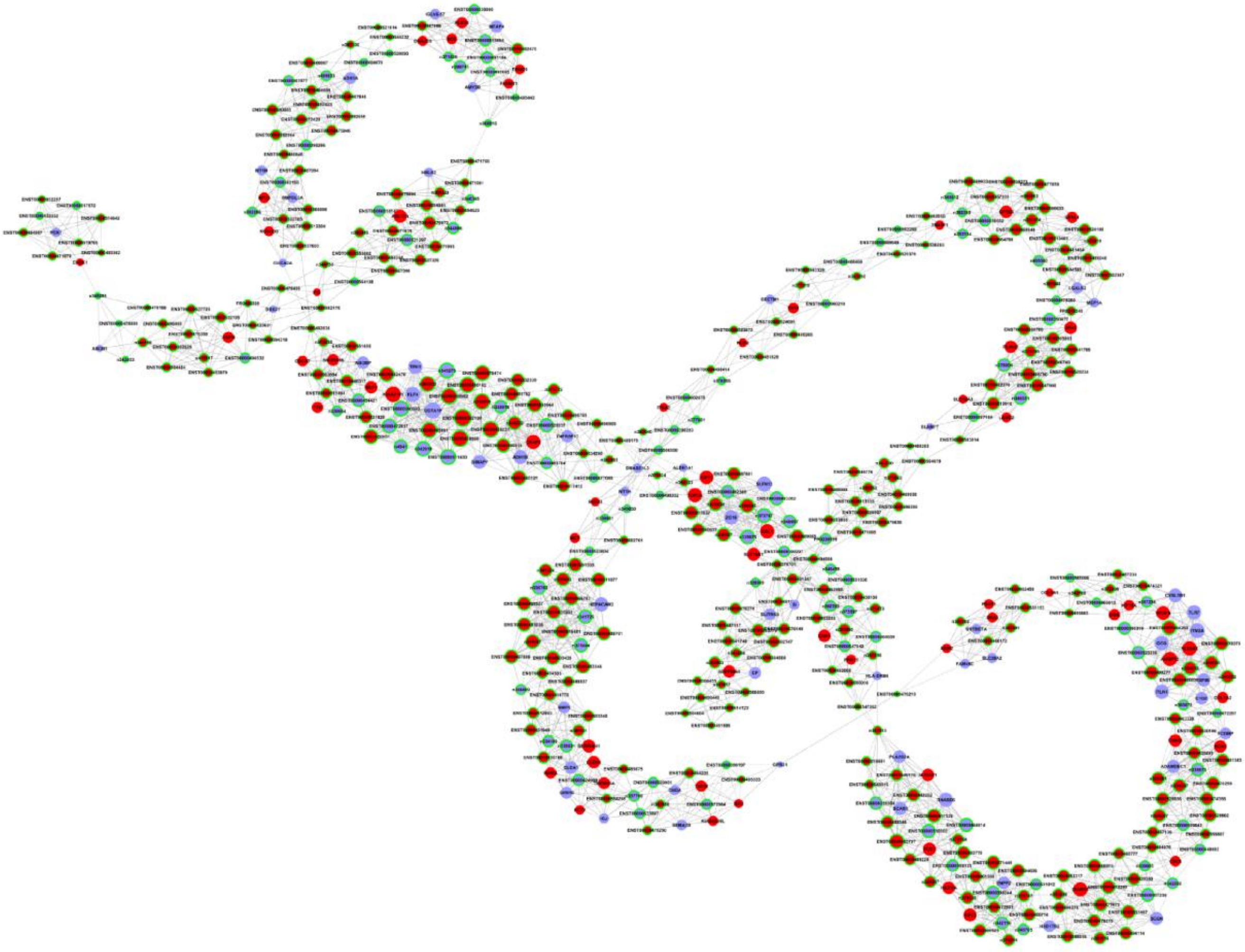

Supplementary Figure 1. lncRNA-mRNA co-expression networks in PT. The mRNA/lncRNA co-expression network in PT. The dots in the figure represent genes, the circled dots represent lncRNA, and the line represents the relationship between the regulation and control element. Solid line represents positive regulation and dashed line represents negative regulation.

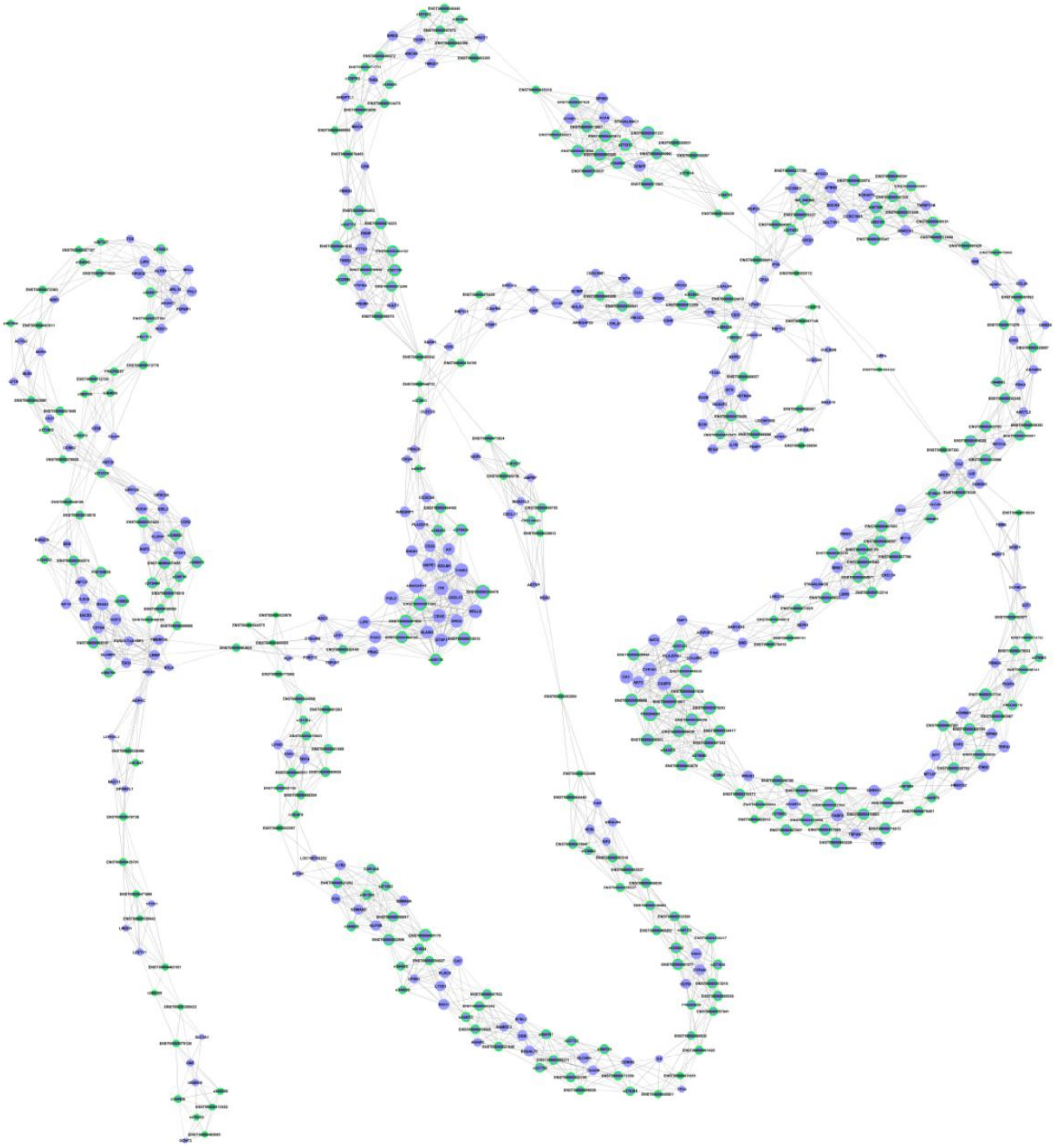

Supplementary Figure 2. lncRNA-mRNA co-expression networks in MLN. The mRNA/lncRNA co-expression network in MLN. The dots in the figure represent genes, the circled dots represent lncRNA, and the line represents the relationship between the regulation and control element. Solid line represents positive regulation and dashed line represents negative regulation.

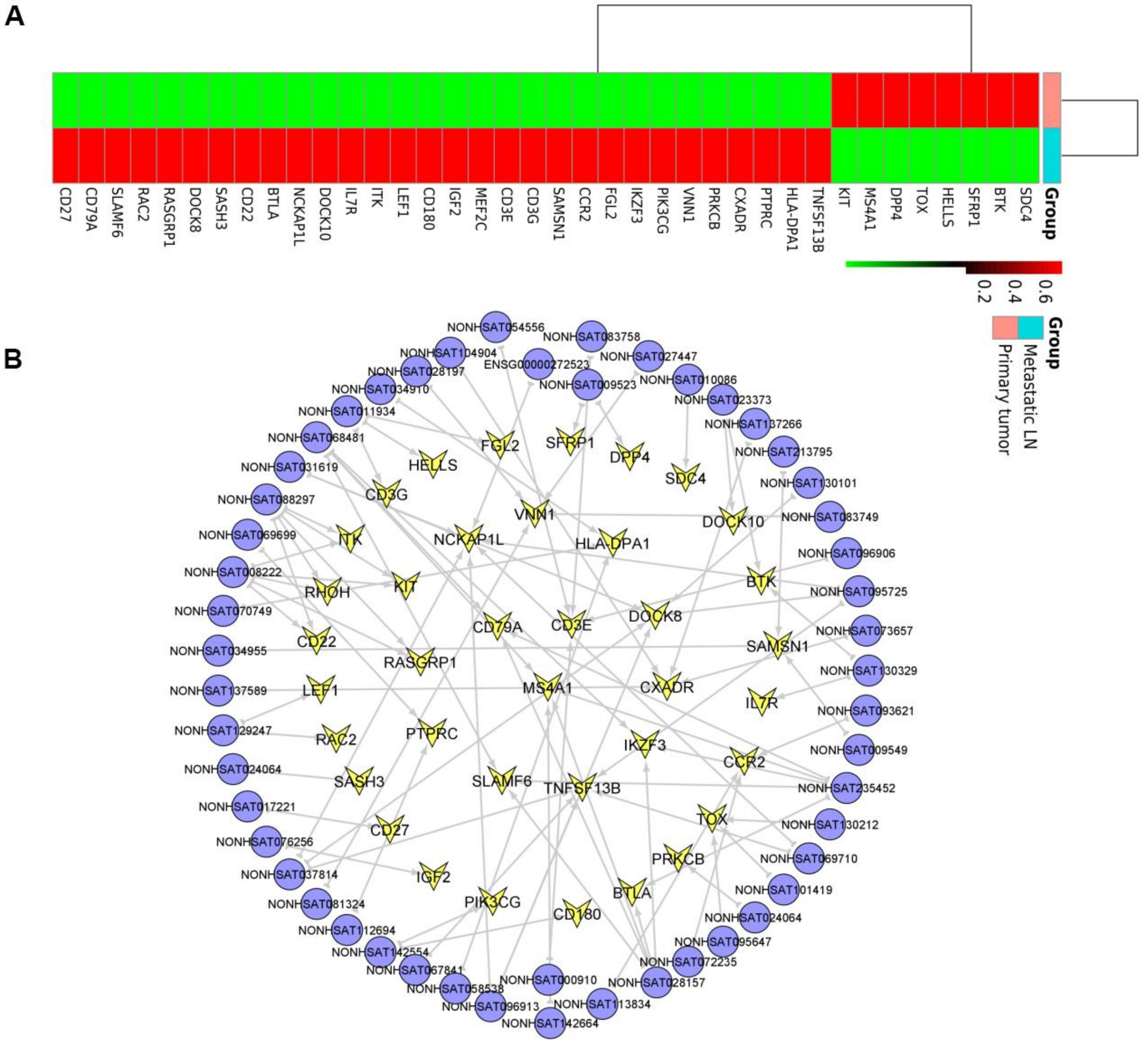

Supplementary Figure 3. Tumor immunity related lncRNAs in CRC. (**A**) The differential lymphocyte activation-related mRNAs in MLN compared with PT. (**B**) lncRNA-mRNA network involved in lymphocyte activation in CRC.

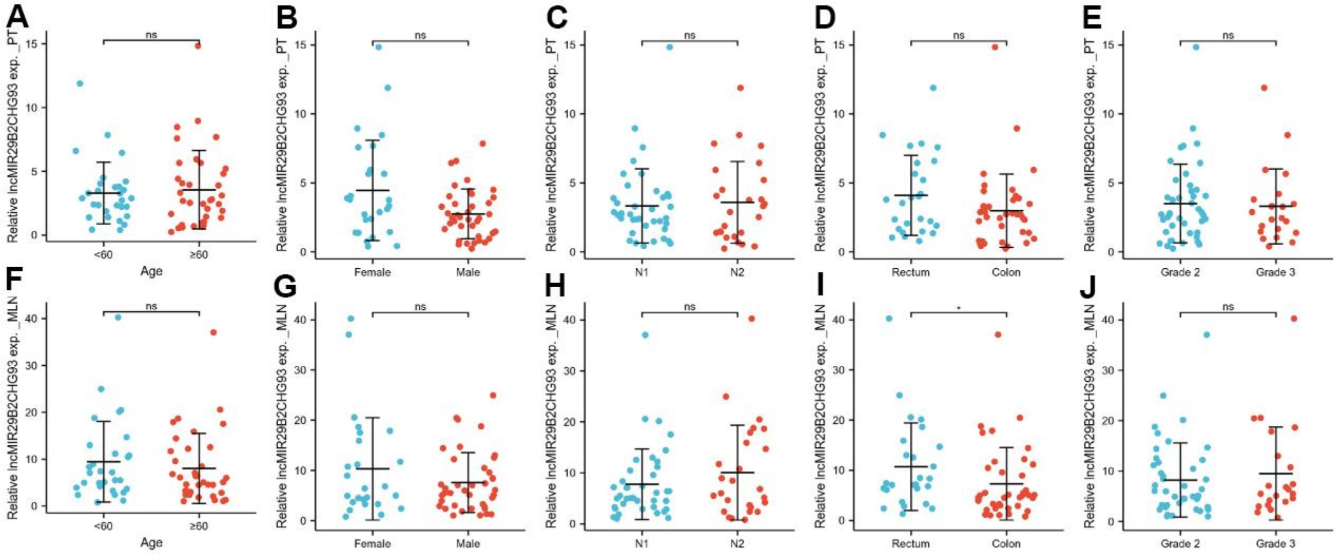

Supplementary Figure 4. The correlation between lncRNA MIR29B2CHG93 expression and clinicopathologic factors in CRC. The lncRNA MIR29B2CHG93 expression in primary tumor tissues from CRC patients. The correlation between expression of lncRNA MIR29B2CHG93 in primary tumor tissue and age (**A**), gender (**B**), lymph node metastasis (**C**), tumor size (**D**) and histological grade (**E**). The lncRNA MIR29B2CHG93 expression in primary tumor tissues from CRC patients. The correlation between expression of lncRNA MIR29B2CHG93 in lymph nodal metastasis tumor tissue and age (**F**), gender (**G**), lymph node metastasis (**H**), tumor size (**I**) and histological grade (**J**). *, P <0.05; **, P <0.01; ***, P <0.001; ****, P <0.0001, respectively. ns denotes no statistical significance. Grade 1, Well differentiated (low grade). Grade 2, Moderately differentiated (intermediate grade). Grade 3, Poorly differentiated (high grade). Grade 4, Undifferentiated (high grade). N0, No regional lymph node metastasis. N1, Metastases in 1 to 3 axillary lymph nodes.
