# Supplementary material for: Identification and functional analysis of lncRNAs and mRNAs between tumorigenesis and metastasis in CRC
Source: Aging (Albany NY). 2021 Dec 26;13(24):25859–85. doi: 10.18632/aging.203775 (PMC8751602; doi:10.18632/aging.203775)
Supplement: Supplementary Tables [file aging-13-203775-s002.pdf]

## SUPPLEMENTARY TABLES

**Supplementary Table 1. Top 10 up and top 10 down differentially expressed lncRNAs in PT vs NM.**

| lncRNAs | Fold-change (PT/NM) | Style | P-value | FDR |
|---|---|---|---|---|
| ENST00000550851 | 12.04 | up | 0.000151 | 0.0031 |
| ENST00000554679 | 10.98 | up | 0.0000988 | 0.00263 |
| ENST00000544679 | 8.44 | up | 0.0008706 | 0.00931 |
| ENST00000497498 | 8.29 | up | 0.0000172 | 0.00105 |
| n345207 | 6.72 | up | 0.0000078 | 0.000656 |
| ENST00000546740 | 6.16 | up | 0.0000045 | 0.000525 |
| ENST00000540885 | 5.58 | up | 0.0001438 | 0.00308 |
| ENST00000497139 | 5.44 | up | 0.0000634 | 0.00209 |
| ENST00000579474 | 5.22 | up | 0.0000304 | 0.00142 |
| n378874 | 5.19 | up | 0.0000864 | 0.00253 |
| n382289 | 27.78 | down | 0.0000002 | 0.0000984 |
| ENST00000424969 | 21.28 | down | 0.0000018 | 0.000354 |
| n334169 | 15.63 | down | 0.0000002 | 0.0000984 |
| ENST00000390319 | 12.20 | down | 0.0000006 | 0.000197 |
| n335875 | 10.99 | down | 0.0000028 | 0.000459 |
| ENST00000390309 | 10.00 | down | 0.0000072 | 0.000656 |
| n383670 | 10.00 | down | 0.000027 | 0.00133 |
| n336760 | 9.09 | down | 0.0000182 | 0.00105 |
| n335821 | 9.09 | down | 0.0000381 | 0.00163 |
| ENST00000572964 | 9.09 | down | 0.0004371 | 0.00582 |

**Supplementary Table 2. Top 10 up and top 10 down differentially expressed lncRNAs in MLN vs NM.**

| lncRNAs | Fold-change (MLN/NM) | Style | P-value | FDR |
|---|---|---|---|---|
| ENST00000554679 | 12.02 | up | 0.0000016 | 0.000796 |
| ENST00000497498 | 10.30 | up | 0.0000028 | 0.000796 |
| ENST00000466541 | 7.03 | up | 0.0000077 | 0.00103 |
| ENST00000550851 | 7.01 | up | 0.0013179 | 0.0137 |
| ENST00000544679 | 6.35 | up | 0.0003233 | 0.00831 |
| ENST00000504484 | 5.91 | up | 0.0000048 | 0.000796 |
| ENST00000579474 | 5.62 | up | 0.0000041 | 0.000796 |
| n332986 | 5.15 | up | 0.0001443 | 0.00737 |
| ENST00000526907 | 4.99 | up | 0.0002169 | 0.00808 |
| ENST00000481038 | 4.45 | up | 0.0113058 | 0.0441 |
| n382289 | 11.90 | down | 0.0000025 | 0.000796 |
| ENST00000424969 | 11.49 | down | 0.000063 | 0.00465 |
| ENST00000560210 | 10.75 | down | 0.0000052 | 0.000796 |
| ENST00000572964 | 10.00 | down | 0.0002002 | 0.00808 |
| n405560 | 7.69 | down | 0.0000187 | 0.002 |
| n368675 | 5.56 | down | 0.005328 | 0.029 |
| n387234 | 5.26 | down | 0.0014105 | 0.0143 |
| ENST00000491695 | 4.76 | down | 0.0000519 | 0.00428 |
| ENST00000491695 | 4.76 | down | 0.0007611 | 0.011 |
| ENST00000486141 | 4.55 | down | 0.0243679 | 0.071 |

**Supplementary Table 3. Top 20 core lncRNAs with high degree in PT.**

| lncRNA | Clustering coefficient | Dgree | Style |
|---|---|---|---|
| ENST00000522190 | 0.66007905 | 23 | up |
| ENST00000478958 | 0.62770563 | 22 | up |
| ENST00000505982 | 0.68571429 | 21 | up |
| n378919 | 0.64761905 | 21 | up |
| ENST00000439231 | 0.63809524 | 21 | up |
| n345275 | 0.82631579 | 20 | down |
| n365932 | 0.82631579 | 20 | up |
| ENST00000465881 | 0.82631579 | 20 | up |
| ENST00000490162 | 0.82631579 | 20 | up |
| ENST00000511430 | 0.82631579 | 20 | down |
| ENST00000390593 | 0.82631579 | 20 | down |
| ENST00000579474 | 0.7 | 20 | up |
| n342918 | 0.85380117 | 19 | down |
| ENST00000495707 | 0.67836257 | 19 | up |
| ENST00000482470 | 0.66666667 | 19 | up |
| n373767 | 0.82352941 | 18 | down |
| n335875 | 0.82352941 | 18 | down |
| n386586 | 0.82352941 | 18 | up |
| ENST00000472617 | 0.81045752 | 18 | down |
| n345687 | 0.77124183 | 18 | up |

**Supplementary Table 4. Top 20 core mRNAs with high degree in PT.**

| Gene symbol | Clustering coefficient | Dgree | Style |
|---|---|---|---|
| GGTA1P | 0.82631579 | 20 | down |
| KIAA0101 | 0.66666667 | 19 | up |
| KLF4 | 0.8496732 | 18 | down |
| SLFN13 | 0.82352941 | 18 | down |
| ITM2A | 0.76470588 | 18 | down |
| TPH1 | 0.91911765 | 17 | down |
| HEPACAM2 | 0.90441176 | 17 | down |
| ZG16 | 0.875 | 17 | down |
| ANGPT2 | 0.81617647 | 17 | up |
| GCG | 0.81617647 | 17 | down |
| SLC6A6 | 0.80882353 | 17 | up |
| KAL1 | 0.78676471 | 17 | up |
| TLR7 | 0.78676471 | 17 | down |
| ITLN1 | 0.77941176 | 17 | down |
| DIAPH3 | 0.75735294 | 17 | up |
| CKAP2 | 0.75 | 17 | up |
| RNASE6 | 0.74264706 | 17 | down |
| TOP2A | 0.67647059 | 17 | up |
| GIMAP7 | 0.79166667 | 16 | down |
| KIF11 | 0.74166667 | 16 | up |

**Supplementary Table 5. Top 20 core lncRNAs with high degree in MLN.**

| lncRNA | Clustering coefficient | Dgree |
|---|---|---|
| ENST00000358476 | 0.72105263 | 20 |
| ENST00000481038 | 0.67894737 | 20 |
| ENST00000551987 | 0.67894737 | 20 |
| FR0204559 | 0.67894737 | 20 |
| ENST00000601390 | 0.78947368 | 19 |
| ENST00000512810 | 0.81699346 | 18 |
| ENST00000491824 | 0.79084967 | 18 |
| ENST00000578243 | 0.71895425 | 18 |
| ENST00000491331 | 0.66013072 | 18 |
| ENST00000449838 | 0.77205882 | 17 |
| ENST00000588806 | 0.75735294 | 17 |
| ENST00000368256 | 0.72794118 | 17 |
| ENST00000479928 | 0.66176471 | 17 |
| ENST00000495178 | 0.52941176 | 17 |
| ENST00000435872 | 0.825 | 16 |
| ENST00000480320 | 0.825 | 16 |
| ENST00000510037 | 0.825 | 16 |
| n344604 | 0.825 | 16 |
| n373212 | 0.825 | 16 |
| ENST00000478698 | 0.80833333 | 16 |

**Supplementary Table 6. Top 20 core mRNAs with high degree in MLN.**

| Gene symbol | Clustering coefficient | Dgree |
|---|---|---|
| ARHGAP15 | 0.7047619 | 21 |
| CXCL13 | 0.7 | 21 |
| ITK | 0.72105263 | 20 |
| GOLM1 | 0.66315789 | 20 |
| CD3G | 0.78947368 | 19 |
| FGL2 | 0.78947368 | 19 |
| FAIM3 | 0.73684211 | 19 |
| CASP5 | 0.71345029 | 19 |
| CYP1B1 | 0.71345029 | 19 |
| NXPE1 | 0.67836257 | 19 |
| GNG2 | 0.82352941 | 18 |
| HELLS | 0.81045752 | 18 |
| GTSF1 | 0.79084967 | 18 |
| CA1 | 0.73856209 | 18 |
| CCDC144A | 0.62091503 | 18 |
| SLAIN1 | 0.80882353 | 17 |
| HLA-DPA1 | 0.77205882 | 17 |
| LIFR | 0.60294118 | 17 |
| AKT3 | 0.8 | 16 |
| NAT2 | 0.8 | 16 |

**Supplementary Table 7. Sequences of the primers used in quantitative RT-PCR.**

| Transcripts | Sequence |
|---|---|
| FN1-227 | F:5′-CCCTGCCAGCTTAAGAACAG-3′<br>R:5′-CCACTTTGCCACTCTCTTCC-3′ |
| lnc-CDR1-1:2 | F:5′-CCGGATAATTTGGGTCTTCC -3′<br>R:5′-GGAGGCCATTGGAAGATGT-3′ |
| lnc-RCHY1-4:1 | F:5′-ATTTCAACCTGGGCAACAAG-3′<br>R:5′-CGGAAGTCTTCAGGGAATCA-3′ |
| SHROOM3-203 | F:5′-CAGTTTCCCTGGTGAAAGGA-3′<br>R:5′-TGTCCATCCATGTCTCTGGA-3′ |
| lnc-RTN4-2:2 | F:5′-GAAAGCACACTTGGCATGAA-3′<br>R:5′-ATGTTTTCAGAGCGGGTCAC-3′ |
| CBX3-208 | F:5′-CTGAGCAGAAACCAAGTTG-3′<br>R:5′-ACCCAAACAACTCACAACG-3′ |
| VCAN-209 | F:5′-TTGAGAAGATGTTGATGAC-3′<br>R:5′-TTATAGCCCTTTTACATTG-3′ |
| LGR5-206 | F:5′-ACCGTCTGCAATCAGTTAC-3′<br>R:5′-ATGGAAGTTGCCTGTTTAC-3′ |
| lnc-ZFYVE26-1:1 | F:5′-CTCATGGAAACAAGCACTG-3′<br>R:5′-TGCCAGATACCTGTCATTG-3′ |
| Lnc-MIR29B2CHG93 | F:5′-ACCAGCATGAATGTGTTCG-3′<br>R:5′-ATCGACACCCAATACATTG-3′ |
| GAPDH | F:5′-GATTCCACCCATGGCAAATTC-3′<br>R:5′-AGCATCGCCCCACTTGATT-3′ |
